# Clinical Significance of Phase Angle in Non-Dialysis CKD Stage 5 and Peritoneal Dialysis Patients

**DOI:** 10.3390/nu10091331

**Published:** 2018-09-19

**Authors:** Byoung-Geun Han, Jun Young Lee, Jae-Seok Kim, Jae-Won Yang

**Affiliations:** Department of Nephrology, Yonsei University Wonju College of Medicine, Wonju, Kang-won 26426, Korea; neptune@yonsei.ac.kr (B.-G.H.); junning@naver.com (J.Y.L.); ripplesong@hanmail.net (J.-S.K.)

**Keywords:** chronic kidney disease, fluid balance, impedance, nutrition, peritoneal dialysis

## Abstract

Background: Fluid overload and protein-energy wasting (PEW) are common in patients with end-stage renal disease (ESRD) and lead to a poor prognosis. We aimed to evaluate the volume and nutritional status of ESRD patients and to determine the clinical significance of phase angle (PhA). Methods: This study was a cross-sectional comparison of bioimpedance spectroscopy (BIS) findings in patients with non-dialysis chronic kidney disease (CKD) stage 5 (CKD5-ND, *N* = 80) and age/sex-matched peritoneal dialysis patients (PD, *N* = 80). PEW was defined as a PhA less than 4.5°. Results: The PhA was found to be positively associated with a geriatric nutritional risk index (GNRI, *r* = 0.561, *p* < 0.001), lean tissue index (LTI, *r* = 0.473, *p* < 0.001), and albumin (*r* = 0.565, *p* < 0.001) while OH/ECW (*r* = −0.824, *p* < 0.001) showed an inverse correlation. The CKD5-ND group had more overhydration (*p* = 0.027). The PD group had significantly higher PhA (*p* = 0.023), GNRI (*p* = 0.005), hemoglobin (*p* < 0.001), and albumin (*p* = 0.003) than the CKD5-ND group. The cut-off values predicting PEW were found to be 3.55 g/dL for albumin, 94.9 for GNRI, and 12.95 kg/m^2^ for LTI in PD patients. Conclusions: This study demonstrated that PhA could be used as a marker to reflect nutritional status in patients with ESRD. Since BIS can inform both volume and nutritional status, regular monitoring will provide the basis for active correction of fluid overload and nutritional supplementation, which may improve outcomes in patients with ESRD.

## 1. Introduction

It is known that 16% to 54% of patients with chronic kidney disease (CKD) are malnourished and that malnutrition itself increases mortality [1]. The protein-energy wasting (PEW) seen in patients with end-stage renal disease (ESRD) is multifactorial and complicated and little is known on this topic. This is because hemodynamic changes, hormonal changes, and persistent inflammatory reactions as well as exacerbation of fluid overload become increasingly complicated as uremia worsens [2]. In addition, a reduction in the nutrient supply due to dietary restrictions causes PEW. Thus, a single modification or intervention treatment may not correct all the factors related to PEW. Occult hypervolemia in patients with CKD is known to cause functional and structural abnormalities to various organs prior to overt hypervolemia, which is another contributing factor to malnutrition.

Simultaneous assessment and management of fluid overload and malnutrition is especially important since it can improve patient outcomes. Therefore, early detection and active treatment with regular monitoring are necessary. Although various methods for assessing nutritional status including clinical symptoms, questionnaires (i.e., subjective global assessment, SGA), several serum laboratory measurements (e.g., serum albumin level), and other novel techniques such as dual energy X-ray absorption spectrometry (DEXA) are available. Each method has limitations [3]. Meanwhile bioimpedance spectroscopy (BIS) performed at the bedside has gained popularity because it is a non-invasive, quick, and relatively affordable method for quantitatively assessing the volume and nutritional status of the patient. The phase angle (PhA) and lean tissue index (LTI) of BIS can also be used to determine a patient’s nutritional status [4,5,6].

We aimed to evaluate the volume and nutritional status of ESRD patients and to determine the clinical significance of PhA in patients with non-dialysis CKD stage 5 (CKD5-ND) and in age/sex-matched peritoneal dialysis patients (PD).

## 2. Materials and Methods

### 2.1. Patients and Data Collection

For the purposes of this study, patients who were in CKD5-ND (pre-dialysis group) (*N* = 80) and patients who had already started PD (PD group) (*N* = 80) at our hospital were included. Initially, 200 patients and 140 patients assigned to the pre-dialysis and PD groups, respectively, among whom age and sex matched 80 patients were randomly selected from each group for analysis. Data on all PD patients were retrieved by reviewing their medical records while the pre-dialysis group were recruited prospectively as a cohort. All CKD5-ND patients were selected from those who were hospitalized to prepare their first dialysis treatment. For the PD group, only the outpatients receiving treatments with PD were considered while inpatients were excluded since only those who have complications during PD treatment would be hospitalized and the condition would have affected the nutritional status as well. Patients diagnosed with malignancy, infection, liver cirrhosis, heart failure, history of renal transplantation, and diagnosed with one kidney were excluded. This study was approved by the Institutional Review Board of Yonsei University Wonju Severance Christian Hospital. All participants provided written informed consent prior to the study.

### 2.2. Assessment of Volume and Nutritional Status

In all patients, BIS was carried out by using the BCM^™^ (Body Composition Monitoring^™^, Fresenius Medical Care, Bad Homburg, Germany), which takes measurements at 50 different frequencies in a range of 5 to 1000 kHz. Parameters obtained directly through BIS were extracellular water (ECW), total body water (TBW), overhydration (OH), and OH/ECW, which reflects the fluid balance. Intracellular water/body weight (ICW/BW), body cell mass (BCM), and PhA were used as markers for a nutritional status. PhA is an angle value of the time delay between the voltage waveform at 50 kHz and the current waveform. The measurement was performed after draining the peritoneal dialysate in PD patients.

BIS and serum measurement were performed at the same time in patients with CKD5-ND and PD. The geriatric nutritional risk index (GNRI) was calculated and used for comparative analysis in all patients [7].

GNRI was calculated using height (H: cm), body weight (BW: kg), and serum albumin level. First, ideal weight (WLo: kg) was calculated according to gender from the Lorentz equations, which is shown below.

Men: WLo = H – 100 − [(H − 150)/4],Women: WLo = H – 100 − [(H − 150)/2.5].Then albumin, ideal weight, and body weight were substituted into the following formula:GNRI = [1.489 × albumin (g/L)] + [41.7 × (BW/WLo)](BW/WLo = 1, when BW exceeded WLo).

### 2.3. Statistical Analysis

All analyses were performed with an IBM Statistics Package for the Social Science version 23.0 (IBM Corporation, Armonk, NY, USA). Descriptive statistics were described as the mean ± standard deviation for continuous variables. Pearson’s correlation analysis was used to examine correlations between PhA and other variables. The Chi-square test and two-sample T-test were used to compare the two groups. To assess independent variables associated with PhA, we performed a multiple linear regression analysis using variables that correlated with PhA at *p* < 0.05 in the univariate analysis: Age, LTI, ICW/BW, OH/ECW, hemoglobin, total protein, and GNRI. GNRI was stratified into four groups [7]. Lastly, we calculated a receiver-operating characteristic (ROC) curve to establish cut-off values of total protein, albumin, GNRI, ICW/BW, and LTI that differentiated patients with PhA > 4.5° from those with PhA ≤ 4.5° in each group. This cut-off was chosen because it has been used in a previous study [8]. Statistical significance was defined as *p* < 0.05.

## 3. Results

### 3.1. Patient Characteristics

The total number of patients was 160 (96 men, 60%). The percentage of diabetic patients in the CKD5-ND group was 58.75% (*N* = 47) and 31.30% (*N* = 25) in the PD group. This percentage difference was significant (*p* < 0.001). The number of fluid overloaded patients (OH/ECW ≥ 15%) was 38 (47.5%) in the CKD5-ND group and 28 (35%) in the PD group. The mean duration of dialysis in patients with PD was 57.51 ± 47.88 months. Among the 80 PD patients, eight were automated peritoneal dialysis patients and 72 were continuous ambulatory peritoneal dialysis patients. Differences between the two groups are shown in Table 1. Age and sex distribution were the same. PD patients had significantly higher PhA and GNRI and lower OH and OH/ECW. The serum parameter that was significantly different between the two groups was albumin (3.42 ± 0.61 g/dL vs. 3.67 ± 0.41 g/dL, *p* = 0.003). However, body mass index (BMI), fat tissue index (FTI), LTI, and BCM were not different.

### 3.2. Univariate Correlation Analysis

PhA was positively associated with GNRI, LTI, BCM, and albumin, while age, OH, and OH/ECW showed an inverse correlation (Table 2). There was also a significant correlation between dialysis duration and PhA in patients with PD (*r* = −0.305, *p* = 0.006).

### 3.3. Multiple Linear Regression Analysis

Multiple linear regression analysis was performed for parameters significantly associated with PhA except OH and albumin due to collinearity. The albumin level was implied in the GNRI value. Age, LTI, OH/ECW, and GNRI 4 were independently associated with PhA, but hemoglobin and total protein were not independently associated. In multivariate linear regression analysis, PhA was significantly associated with OH/ECW (standardized regression coefficient (β = −0.690, *p* < 0.001), LTI (β = 0.265, *p* < 0.001), age (β = −0.159, *p* < 0.001), GNRI4 (β = 0.152, *p* = 0.037), and ICW/BW (β = 0.103, *p* < 0.001) (Table 3).

### 3.4. Cut-Off Values of Nutritional Markers

ROC curves were drawn for the total protein, albumin, GNRI, ICW/BW, and LTI to determine the cut-off values predicting PhA greater than 4.5° (Table 4).

## 4. Discussion

In general, chronic disease with malnutrition is related to poor prognosis. While hypoalbuminemia is not a synonym for malnutrition, it is associated with morbidity and mortality [9,10]. Malnutrition in ESRD patients can be multifactorial and complicated. This is a difficult issue in monitoring and treating malnourished patients. In patients undergoing dialysis, it is unclear whether hypoalbuminemia is simply secondary to poor nutrition itself or is associated with other complex conditions such as chronic systemic inflammation, fluid overload, underlying comorbidities, and impaired compensatory hepatic synthesis of albumin [11].

Increased albumin turnover in peritoneal dialysis patients with ongoing losses of protein into dialysate results in hypoalbuminemia, which is the result of chronic inflammation rather than malnutrition [12,13]. Peritoneal protein losses have not predicted outcomes in PD patients [14]. Inflammatory states can affect the serum level of albumin [15]. Albumin as a negative acute-phase reactant during inflammation is generally independent of nutritional intake [16]. This means that there is a limit to albumin as a marker of true malnutrition and interpretation of the medical implications of low serum albumin in PD patients. GNRI is calculated by using height, body weight, and serum albumin level. Although GNRI was proposed as a strong predictor of overall mortality in hemodialysis patients, it has not been a sensitive tool for screening malnutrition in PD patients [17,18]. Therefore, regarding the therapeutic approach to malnourished patients, evaluation items other than albumin are needed in order to complement the weakness of albumin [13].

Fluid overload is one predictor of mortality in ESRD patients and is closely related to adverse cardiovascular outcomes [19,20,21]. The volume status may independently affect serum albumin. Dilutional hypoalbuminemia induced by fluid overload can be seen in ESRD patients. In our study, the CKD5-ND group was more likely to show overhydration when compared to the PD group. This seems to suggest that intervention stabilizes the fluid balance of PD patients. The body mass index (BMI) does not discriminate between lean mass, fat, and water. Therefore, the use of BMI alone has limitations when assessing the nutritional status of these patients because it can hide PEW in overhydrated individuals. PEW is common in ESRD patients. Regarding the diagnosis of PEW in ESRD, the potential usefulness of nutritional markers has not been completely established. PEW was inversely assessed by the ICW/BW [22]. Serum albumin is one of the definition criteria for the diagnosis of PEW [23]. It has been noted that the combined utilization of several markers is preferable for nutrition rather than a single marker since PEW markers appear to be more useful for a nutritional status assessment in hemodialysis patients. Moreover, PhA has been significantly associated with PEW when evaluated by combined criteria [24].

PhA has been widely used as a nutritional assessment tool in patients with liver cirrhosis, colon cancer, head and neck cancer, and other conditions [25,26,27]. It can also reflect a nutritional status in ESRD patients undergoing maintenance hemodialysis or not on dialysis [28]. PhA can predict arterial stiffness and vascular calcification in stable PD patients [29]. PhA was also a predictor of clinical outcomes in dialysis patients and patients not yet on dialysis [30,31,32]. Patients with PD had a higher PhA than hemodialysis patients [33]. However, there is no consensus about the appropriate cut-off value for PhA in determining or predicting outcomes. Moreover, PhA has been used in studies of other diseases but rarely used in studying CKD5 patients.

In the current study, hemoglobin, albumin, GNRI, and PhA were higher in PD patients with low uremic toxin levels and less fluid overload compared to the CKD5-ND patients. Low serum albumin, which is one of the chemical criteria for PEW, appears to have a higher PEW in CKD5-ND patients. Herein, we need to know that evaluating PD patients who receive a fraction of their energy requirement from glucose-containing dialysis fluid using a single marker can mask results such as a loss of muscle mass. It is not clear which method provides the most reliable method of assessing the nutritional status of ESRD patients. Therefore, it is desirable to evaluate the clinical results in a comprehensive manner using reliable, easy, and affordable methods [3,13].

This study had some limitations. First, this was a single-center study that included a relatively small number of patients. Inflammatory markers were not measured and SGA was not evaluated. The amount of protein lost by the dialysate was not evaluated. There was a difference in the proportion of diabetics between the two groups. We could not match the cause of disease between the two groups and could not match patients’ comorbidities. All patients received renal nutritional recommendations. However, we did not control their dietary intake except in special cases such as hyperkalemia. Lastly, the residual renal function or total clearance of PD patients could not be measured. Despite these limitations, the strength of this study is that we objectively measured the nutritional status of patients who were not receiving dialysis treatment and those who were receiving peritoneal dialysis as well as their fluid balance. As far as we know, this is the first study to have attempted such an analysis.

## 5. Conclusions

Our findings show that suitable assessment tools for nutritional and volume status are needed and can serve as clues for early interventions for malnourishment and fluid overload in this patient group. Since BIS can inform both volume and nutritional status, regular monitoring will provide the basis for active correction of fluid overload and nutritional supplementation. However, larger prospective studies are needed in order to determine the clinical significance of PhA in patients with ESRD.

## Figures and Tables

**Table 1:** Comparison of the CKD5-ND group and PD group.

Variables	Total (*N* = 160)	CKD5-ND	PD	*p*-Value
		(*N* = 80)	(*N* = 80)	
Age, years	56.93 ± 9.89	56.93 ± 9.89	56.93 ± 9.89	1.000
Phase angle, °	4.70 ± 1.29	4.47 ± 1.37	4.93 ± 1.16	0.023
GNRI	93.66 ± 8.27	91.84 ± 9.40	95.47 ± 6.54	0.005
BMI, kg/m^2^	24.69 ± 3.80	25.21 ± 4.10	24.17 ± 3.47	0.083
BCM, kg	21.45 ± 6.69	21.42 ± 6.86	21.48 ± 6.57	0.949
ICW/BW, L/kg	0.28 ± 0.05	0.28 ± 0.05	0.28 ± 0.05	0.314
LTI, kg/m^2^	14.36 ± 3.10	14.42 ± 3.32	14.31 ± 2.90	0.810
FTI, kg/m^2^	9.10 ± 4.57	9.24 ± 4.89	8.98 ± 4.25	0.719
Hemoglobin, g/dL	9.78 ± 1.51	9.15 ± 1.43	10.40 ± 1.31	<0.001
Total Protein, g/dL	6.32 ± 0.74	6.11 ± 0.79	6.54 ± 0.62	<0.001
Albumin, g/dL	3.55 ± 0.53	3.42 ± 0.61	3.67 ± 0.41	0.003
Total cholesterol, mg/dL	160.15 ± 49.14	155.78 ± 58.71	164.53 ± 37.08	0.261
BUN, mg/dL	73.04 ± 25.17	90.07 ± 21.53	56.02 ± 15.00	<0.001
Uric acid, mg/dL	7.82 ± 1.86	8.43 ± 2.24	7.26 ± 1.19	<0.001
OH, Liter	2.83 ± 3.82	3.67 ± 4.85	1.99 ± 2.10	0.005
OH/ECW, %	13.91 ± 15.03	16.53 ± 17.89	11.29 ± 10.99	0.027
ECW, Liter	17.06 ± 4.55	17.86 ± 5.55	16.26 ± 3.11	0.026
ICW, Liter	17.97 ± 3.93	17.91 ± 3.94	18.02 ± 3.94	0.868
TBW, Liter	35.02 ± 7.47	35.77 ± 8.35	34.27 ± 6.43	0.205

BCM, body cell mass. BMI, body mass index. BUN, blood urea nitrogen. BW, body weight. CKD5-ND, non-dialysis chronic kidney disease stage 5. ECW, extracellular water. FTI, fat tissue index. GNRI, geriatric nutritional risk index. ICW, intracellular water. LTI, lean tissue index. OH, overhydration. PD, peritoneal dialysis. TBW, total body water.

**Table 2:** Univariate analysis of nutritional markers, serum laboratory tests, and volume markers in association with the phase angle.

Variables	Phase Angle	
	Correlation Coefficient	*p*-Value
Age, years	−0.239	0.002
GNRI	0.561	<0.001
BMI, kg/m^2^	−0.036	0.654
BCM, kg	0.482	<0.001
ICW/BW, L/kg	0.502	<0.001
LTI, kg/m^2^	0.473	<0.001
FTI, kg/m^2^	−0.102	0.201
Hemoglobin, g/dL	0.255	0.001
Total Protein, g/dL	0.492	<0.001
Albumin, g/dL	0.565	<0.001
Total cholesterol, mg/dL	−0.194	0.014
BUN, mg/dL	0.035	0.662
OH, Liter	−0.738	<0.001
OH/ECW, %	−0.824	<0.001
ECW, Liter	−0.370	<0.001
ICW, Liter	0.484	<0.001
TBW, Liter	0.028	0.721

BCM, body cell mass. BMI, body mass index. BUN, blood urea nitrogen. BW, body weight. ECW, extracellular water. FTI, fat tissue index. GNRI, geriatric nutritional risk index. ICW, intracellular water. LTI, lean tissue index. OH, over-hydration. TBW, total body water.

**Table 3:** Multiple linear regression analysis of variables influencing the phase angle (*N* = 160).

	Unstandardized Coefficients		Standardized Coefficients	*p*-Value
	B	Standard Error	β	
Age	−0.021	0.004	−0.159	<0.001
LTI, kg/m^2^	0.110	0.020	0.265	<0.001
ICW/BW, L/kg	2.640	1.253	0.103	<0.001
OH/ECW, %	−5.902	0.307	−0.690	<0.001
Hemoglobin, g/dL	0.022	0.026	0.026	0.395
Total protein, g/dL	0.080	0.077	0.046	0.302
GNRI 1 (<82)	reference			
GNRI 2 (82−92)	0.041	0.152	0.014	0.789
GNRI 3 (92−98)	0.275	0.163	0.097	0.093
GNRI 4 (>98)	0.409	0.187	0.152	0.037

Dependent variable: phase angle. Independent variables: age, LTI, ICW/BW, OH/ECW, hemoglobin, total protein, GNRI. A significant regression equation was found (F(9, 150) = 120.09, *p* < 0.001) with an *R*^2^ of 0.878. B, unstandardized regression coefficient. β, standardized regression coefficient. BW, body weight. ECW, extracellular water. GNRI, geriatric nutritional risk index. ICW, intracellular water. LTI, lean tissue index. OH, overhydration.

**Table 4:** Cut-off values of parameter indices by ROC curve analysis in each group.

	Cut-Off Value	AUC	*p*-Value	Sensitivity (%)	Specificity (%)
CKD5-ND					
Total Protein, g/dL	6.05	0.7679	<0.001	70.59	71.74
Albumin, g/dL	3.45	0.8082	<0.001	76.47	71.74
GNRI	92.83	0.8027	<0.001	76.47	69.57
ICW/BW, L/kg	0.2696	0.6953	0.003	64.71	69.57
LTI, kg/m^2^	13.85	0.6752	0.008	64.71	73.91
PD					
Total Protein, g/dL	6.30	0.7285	<0.001	59.38	77.08
Albumin, g/dL	3.55	0.7630	<0.001	62.50	85.42
GNRI	94.90	0.7692	<0.001	68.75	79.17
ICW/BW, L/kg	0.2687	0.7415	<0.001	71.88	75.00
LTI, kg/m^2^	12.95	0.8148	<0.001	68.75	85.42

ROC, receiver-operating characteristic. AUC, area under the curve. BW, body weight. CKD5-ND, non-dialysis CKD stage 5. GNRI, geriatric nutritional risk index. ICW, intracellular water. LTI, lean tissue index. PD, peritoneal dialysis.
